# Three new species of *Helicogloea* (Phleogenaceae, Atractiellales) from South China

**DOI:** 10.3897/mycokeys.132.188722

**Published:** 2026-05-08

**Authors:** Zi-Wei Zheng, Qian-Xin Guan, Shu-Bin Liu, Fang Wu

**Affiliations:** 1 State Key Laboratory of Efficient Production of Forest Resources, School of Ecology and Nature Conservation, Beijing Forestry University, Beijing 100083, China State Key Laboratory of Efficient Production of Forest Resources, School of Ecology and Nature Conservation, Beijing Forestry University Beijing China

**Keywords:** New taxa, phylogeny, taxonomy, wood-decaying fungi

## Abstract

Three new species of *Helicogloea* from south China, *Helicogloea candida*, *H. conidiata* and *H. sinosebacea*, are illustrated and described, based on morphological characteristics and multigene phylogenetic analyses of the internal transcribed spacer regions (ITS) and the large subunit of the nuclear ribosomal RNA gene (nLSU). *Helicogloea candida* is characterised by resupinate, gelatinous, hyaline to whitish, first pustulate to slightly cerebriform when fresh and ellipsoid to oblong ellipsoid basidiospores measuring 8.9–13.6 × 4.4–8.9 μm; *H. conidiata* is characterised by the presence of a sporodochial asexual morph and subglobose to ellipsoid conidia measuring 3.6–4.5 × 2.9–3.8 µm; *H. sinosebacea* is characterised by resupinate, gelatinous, whitish or greyish basidiomes when fresh and subglobose to broadly ellipsoid basidiospores measuring 7–10 × 4.8–7.3 μm. The differences amongst the new species and their morphologically similar and phylogenetically related species are discussed.

## Introduction

The genus *Helicogloea* Pat. (Atractiellales, Atractiellomycetes, Basidiomycota), typified by *H. lagerheimii* Pat., is characterised by semi-translucent, waxy or gelatinous basidiomes, hyphae without clamp connections, probasidia with a lateral probasidial sac, in which karyogamy occurs and segmented hypobasidia with the segments in linear series, basidia normally four-celled, transversally septate and usually cylindrical to broadly ellipsoid or subglobose basidiospores (Patouillard and Lagerheim 1892; Baker 1936, 1946; Spirin 1936; Spirin et al. 2018; Malysheva et al. 2020; Liu et al. 2025). In addition, sporodochial asexual morphs have been detected in *Helicogloea* species, such as *H. sebacea* (Bourdot & Galzin) Spirin & Trichies (Kirschner 2004; Spirin et al. 2018). The species of *Helicogloea* are saprotrophic on decaying wood and various plant debris (Malysheva et al. 2020).

The genus *Helicogloea* was established by Patouillard to accommodate a single species, *H. lagerheimii*, from South America (Patouillard and Lagerheim 1892). Möller (1895) proposed the genus *Helicogloea* as a synonym of *Saccoblastia* Möller and emphasised the importance of saccate probasidia as the main generic feature. Thereafter, Baker (1936) confirmed that the type species, *H. lagerheimii*, shared the saccate probasidia with species of *Saccoblastia* and reinstated *Helicogloea* to replace *Saccoblastia* because of the older epithet *Helicogloea*. Donk (1966) proposed to restrict *Helicogloea* to include only gelatinous species and *Saccoblastia* to include those species with floccose, non-hygroscopic basidiocarps. Aime et al. (2006) and Bauer et al. (2006) demonstrated that *Helicogloea* and *Saccoblastia* were not congeneric and clustered into different lineages within Atractiellomycetes in phylogenetic analyses. In recent years, the genus *Helicogloea* has attracted increasing attention from researchers. Based on morphological and molecular data, Spirin et al. (2018) revised the taxonomy of the *Helicogloea* and described eleven new species of *Helicogloea*. Malysheva et al. (2020) described two new species of *Helicogloea* from the Nordic Region and Liu et al. (2025) reported a new species, *H. hangzhouensis* F. Wu et al. from China, based on morphological and molecular data. Currently, 47 names of *Helicogloea* are recorded in Index Fungorum (http://www.indexfungorum.org, accessed 9 Apr 2026) and around 34 species are accepted and legitimate, but only 18 species were confirmed by molecular data (Malysheva et al. 2020; Liu et al. 2025). Geographically, *Helicogloea* species have been widely reported in Asia, Europe, North America and South America, occasionally in Europe and South America (Spirin et al. 2018; Malysheva et al. 2020; Liu et al. 2025).

During an investigation of wood-rotting fungi in south China, nine *Helicogloea* specimens were collected; morphologically, they can be distinguished from known *Helicogloea* species and, phylogenetically, they form three distinct new lineages. Therefore, they are described and illustrated as three new species of *Helicogloea*.

## Materials and methods

### Morphological studies

The studied specimens were collected from Guangxi Autonomous Region, Yunnan, Zhejiang and Hubei Province in south China. The fruiting bodies were photographed *in situ*, collection details were recorded (Rathnayaka et al. 2025) and the fruiting bodies were taken to the laboratory in plastic collection boxes. They were deposited at the Herbarium of the Institute of Microbiology, Beijing Forestry University (BJFC). Macro-morphological descriptions were based on field notes and dried specimens. Microscopic features were examined and described in 5% KOH (potassium hydroxide) and 2% phloxine B (C_20_H_2_Br_4_Cl_4_Na_2_O_5_) with a magnification of up to 1,000 × using a Nikon Eclipse 80i microscope and phase contrast illumination. Colour terms followed Anonymous (1969) and Petersen (1996). A Nikon Digital Sight DS-L3 camera was used to photograph microscopic structures. Thirty basidiospores from each specimen were measured. The following abbreviations were used: L = arithmetic average of basidiospores length, W = arithmetic average of basidiospores width, Q = L/W ratios, (n = x/y) = the number of spores (x) measured from a given number of specimens (y).

### DNA extraction, PCR amplification and sequencing

A cetyl trimethylammonium bromide (CTAB) rapid plant genome extraction kit (Aidlab Biotechnologies, Co., Ltd., Beijing, China) was used to obtain DNA products from voucher specimens following the manufacturer’s instructions with some modifications (Wu et al. 2020, 2022; Wang et al. 2024; Song et al. 2025). The following primer pairs were used to amplify the DNA: ITS5 and ITS4 for the internal transcribed spacer (ITS) region (White et al. 1990) and LR0R and LR7 for the nuclear large subunit (nLSU) rDNA gene (Vilgalys and Hester 1990).

The PCR procedure for ITS was as follows: initial denaturation at 95 °C for 3 min, followed by 35 cycles at 94 °C for 40 s, 54 °C for 45 s and 72 °C for 1 min, with a ﬁnal extension of 72 °C for 10 min. The PCR procedure for nLSU was as follows: initial denaturation at 94 °C for 1 min, followed by 35 cycles at 94 °C for 30 s, 50 °C for 1 min and 72 °C for 1.5 min, with a ﬁnal extension of 72 °C for 10 min (Dong et al. 2024; Yang et al. 2025; Cui et al. 2026). The PCR products were purified and sequenced by Beijing Genomics Institute (BGI), China. All newly-generated sequences in this study were deposited in GenBank (http://www.ncbi.nlm.nih.gov/genbank/; Sayers et al. (2024)) and listed in Table 1.

**Table 1.** Names, voucher numbers, countries and corresponding GenBank numbers of the taxa used in the phylogenetic analyses of *Helicogloea*.

Species	Voucher number	Country	GenBank accession number	
			ITS	nLSU
*Atractidochium hillariae*	caw 010	–	KM519231	–
*A. hillariae*	caw 011	–	KM519232	–
*Bourdotigloea cerea* *	VS 11057	Norway	MH304504	MH304455
*B. concisa* *	GT 11015	France	MH304505	MH304456
*Helicogloea aquilonia*	Söderholm 4241	Finland	MH304476	–
*H. aquilonia* *	VS 11163	Russia	MH304479	MH304440
*H. aquilonia*	Degelius w/n	Norway	MH304478	MH304439
*H. aseptata* *	Spirin 12172	Russia	MK880145	MK880143
*H. burdsallii* *	CFMR HHB-6017	USA	MH304484	–
H. candida*	**Wu 4197**	**Zhejiang, China**	**PX993294**	**–**
***H. candida***	**Wu 2967**	**Guangxi, China**	**PX993292**	**PX993301**
***H. candida***	**Wu 2973**	**Guangxi, China**	**PX993293**	**PX993302**
***H. candida***	**Wu 2924**	**Yunnan, China**	**PX993295**	**–**
*H. compressa*	LE 313253	Russia	MH304482	MH304442
*H. compressa*	TU119718	USA	MH304481	MH304441
*H. compressa*	OM 19493	USA	MH304480	–
H. conidiata*	**Wu 2813**	**Yunnan, China**	**PX993290**	**PX993299**
***H. conidiata***	**Wu 2814**	**Yunnan, China**	**PX993291**	**PX993300**
*H. crassitexta* *	LE 312773	Russia	MH304483	MH304443
*H. dryina*	JN 7745	Sweden	MH304486	MH304444
*H. dryina* *	SS 786	Norway	MH304485	–
*H. eburnea* *	AS 171127/1127A	Kenya	MH304487	MH304445
*H. exigua* *	CFMR HHB-8162	USA	MH304488	MH304446
H. hangzhouensis*	Wu 642	Zhejiang, China	OR515653	OR515656
*H. hangzhouensis*	Wu 652	Zhejiang, China	OR515654	OR515657
*H. lagerheimii*+	KW 1711-2	USA	MH304489	–
H. lunula*	PDD 88360	New Zealand,	MH304490	–
H. microsaccata*	LE 262936	Russia	MH304491	–
H. pellucida*	VS 10610	Russia	MH304493	MH304448
*H. pellucida*	CWU 4000	Ukraine	MH304492	MH304447
*H. sebacea*	JHC 11-066	Denmark	MH304495	–
*H. sebacea*	GT 07024	France	MH304494	–
*H. sebacea*	CWU-6331	Ukraine	MH304502	–
*H. septifera*	VS 11043	Norway	MH304499	MH304452
*H. septifera*	LE 260	Russia	MH304497	MH304450
*H. septifera*	LE 253866	Russia	MH304496	MH304449
***H. sinosebacea***	**Wu 3224**	**Zhejiang, China**	**PX993289**	**PX993298**
***H. sinosebacea***	**Wu 4211**	**Zhejiang, China**	**PX993287**	**PX993296**
H. sinosebacea*	**Wu 4611**	**Hubei, China**	**PX993288**	**PX993297**
*H.* sp.	RJB 6478-5	–	MF476085	–
*H.* sp.	KW 3141	Canada	MH304503	–
*H. sputum* *	O F90728	Denmark	MH304500	MH304453
*H. subardosiaca* *	JP 3668	Finland	MH304501	MH304454
*Saccosoma farinaceum*	VS 11092	Norway	MH304471	MH304432

Notes: Newly-generated sequences are in bold; “–” represents missing data; “*” represents type specimens. “+” represents generic type.

### Phylogenetic analyses

The newly-generated sequences were checked for ambiguous bases and assembled using BioEdit. The new and reference sequences retrieved from GenBank through BLAST searches (Table 1) were partitioned into ITS1, 5.8S, ITS2 and nLSU. *Saccosoma farinaceum* (Höhn.) Spirin & K. Põldmaa was used as the outgroup (Spirin et al. 2018; Liu et al. 2025). These sequences were aligned with MAFFT v.7.526 (http://mafft.cbrc.jp/alignment/server/; Katoh et al. (2019)) and then manually adjusted in BioEdit and Mesquite version 3.04 (Hall 1999; Maddison and Maddison 2017). The separate alignments were then concatenated using Mesquite v.3.70. The final alignments and the retrieved topologies were deposited in TreeBASE (http://www.treebase.org) under accession 32513.

Maximum Likelihood (ML) analysis was conducted using the RaxML-HPC BlackBox tool via the CIPRES Science Gateway (Miller et al. 2010). The maximum run-time for the RaxML half-bootstrap analysis was set to the default of 0.25 hours (15 minutes), with all other parameters retained at their defaults.

Bayesian Inference (BI) analysis was performed using MrBayes v.3.2.7 (Ronquist et al. 2012). The best-fit partitioning scheme and substitution models were determined using the “greedy” algorithm in ModelFinder, with branch lengths estimated as “linked” under the AICc criterion (Kalyaanamoorthy et al. 2017). Two independent runs were executed, each with four Markov chains (including one cold chain), starting from random trees and continuing for one million generations until the average standard deviation of split frequencies fell below 0.01. Trees were sampled every 1000 generations. The first 25% of sampled trees were discarded as burn-in and the remaining trees were used to construct a majority-rule consensus tree and calculate Bayesian posterior probabilities (BPP) for the clades.

Phylogenetic trees were visualised using FigTree version 1.4.4 (Rambaut 2018). Branches were considered significantly supported if they simultaneously met the following criteria: ML bootstrap support (BS) ≥ 70% and BPP ≥ 0.95.

## Results

### Phylogenetic analyses

**Figure 1.**
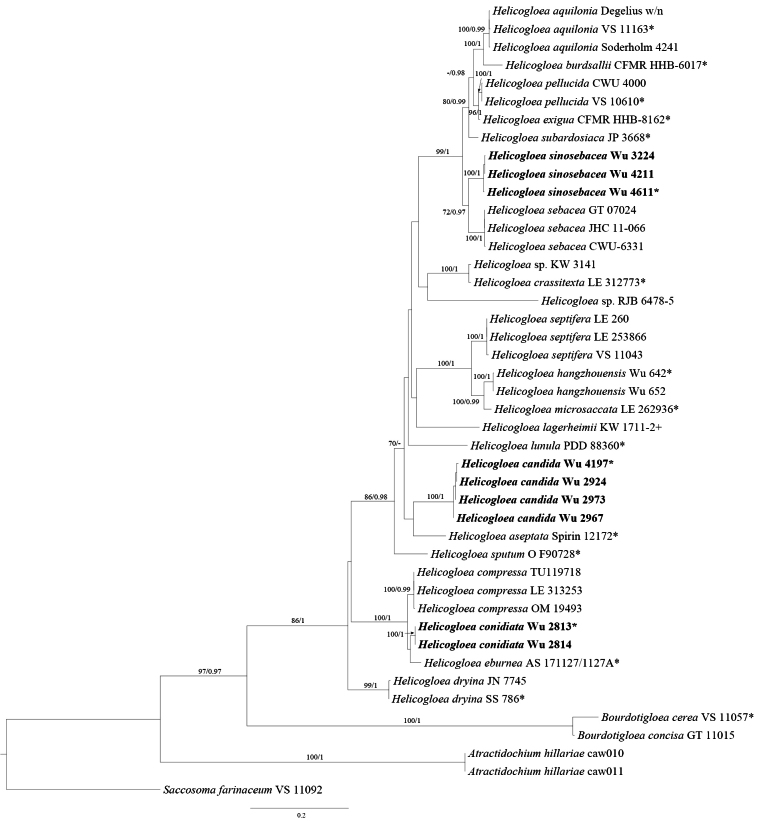
Maximum Likelihood (ML) tree illustrating the phylogeny of *Helicogloea*, based on a combined ITS1-5.8S-ITS2-nLSU dataset. Branches are labelled with parsimony bootstrap values (ML) equal to or higher than 70% and Bayesian Posterior Probabilities (BPPs) equal to or higher than 0.95. *Saccosoma farinaceum* was selected as the outgroup. “*” indicates the type specimens. “+” indicates the generic type.

In this study, the combined ITS1-5.8S-ITS2-nLSU dataset included sequences from 44 specimens, representing 26 species of *Helicogloea* and *Saccosoma farinaceum* as the outgroup (Fig. 1). ModelFinder proposed the models SYM+G4 for ITS1, K2P+G4 for 5.8S, HKY+F+G4 for ITS2 and GTR+F+G4 for nLSU for the Bayesian analysis. The BI analysis yielded an average standard deviation of split frequencies of 0.003227.

The ML and BI analyses resulted in nearly identical topologies and only the ML tree is presented with the bootstrap supports for ML and BPP not less than 70% and 0.95, respectively (Fig. 1). Our phylogenies, based on Bayesian and ML, demonstrated that our specimens formed three new lineages with high support (100/1, 100/1, 100/1, respectively). Amongst them, Wu 3224, Wu 4211 and Wu 4611 formed a strong support lineage (100/1.00), namely *H. sinosebacea*, closely related to *H. sebacea*. Wu2924, Wu 2967, Wu 2973, and Wu 4197 formed an independent, well-supported lineage (100/1.00), namely *H. candida*, which is most closely related to *H. aseptata* Malysheva & Spirin. Wu 2813 and Wu 2814 formed a high-support lineage (100/1.00), namely *H. conidiata*, grouped with *H. compressa* (Ellis & Everh.) Malysheva & K. Põldmaa and *H. eburnea* A. Savchenko & Malysheva.

### Taxonomy

#### Helicogloea candida

Taxon classificationFungiAtractiellalesPhleogenaceae

Z.W. Zheng, Q.X. Guan & F. Wu sp. nov.

3C56CD0E-1E2C-5208-B4F1-7CB7C1448CA4

MB862396

Figs 2, 3

##### Holotype.

China • Zhejiang Province, Hangzhou, Linan County, West Tianmu Mountain, 30°19'45"N, 119°26'19"E, 510 m a.s.l., 14 September 2025, on rotten angiosperm wood, F. Wu leg., Wu 4197 (BJFC064313, holotype).

**Figure 2.**
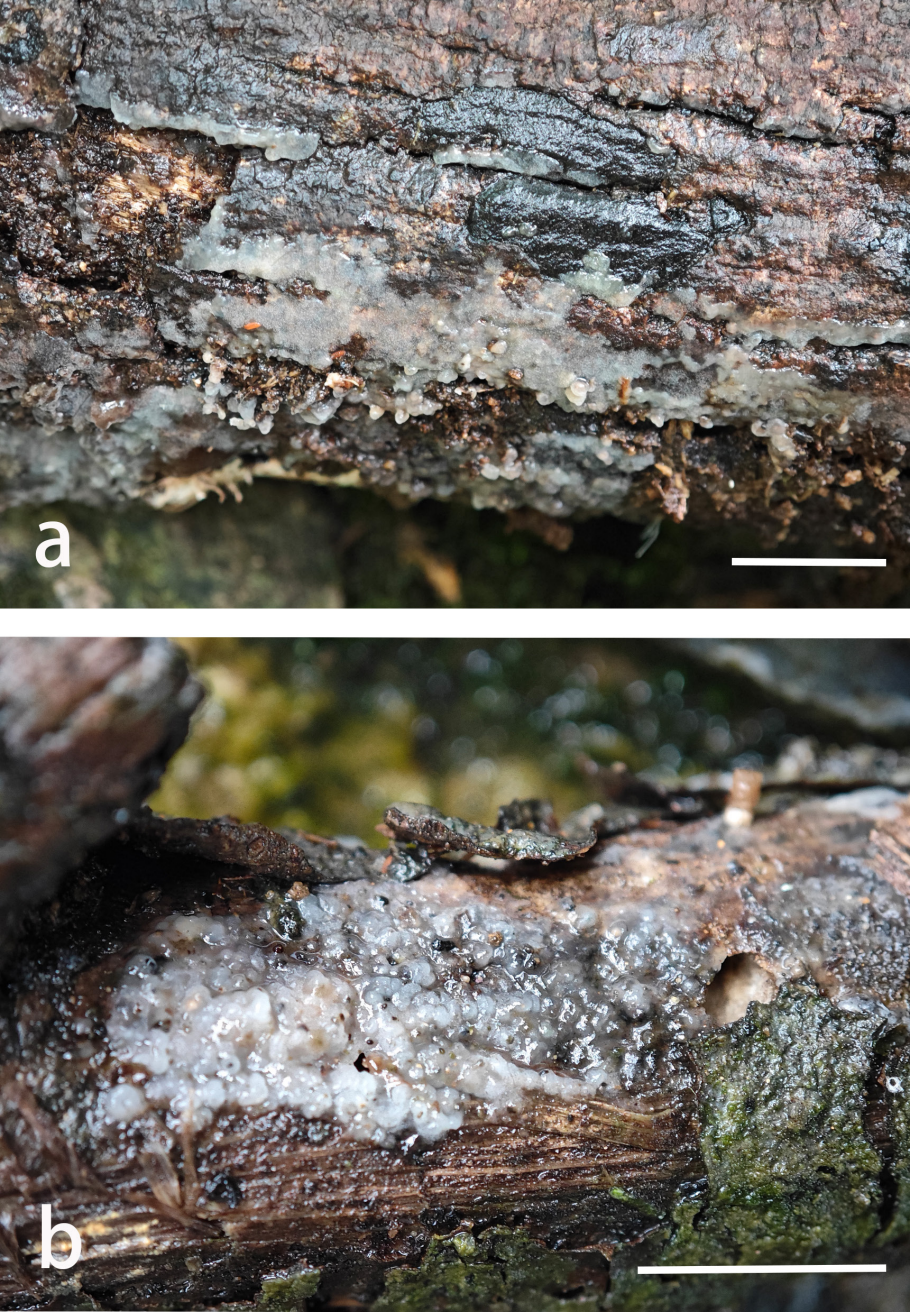
Basidiomes of *Helicogloea candida*. **a**. Wu 4197 (holotype); **b**. Wu 2924. Scale bars: 1 cm (**a, b**).

##### Etymology.

*Candida* (Lat.): refers to the new species having whitish basidiomes.

##### Description.

Basidiomes annual, resupinate, gelatinous when fresh, hyaline to whitish, first pustulate to slightly cerebriform, then fusing together and turning semi-translucent and waxy, hymenial surface often tuberculate, up to 4 cm long, 1 cm wide and 0.1 cm thick, almost invisible when dry. Hyphal system monomitic, simple septate, hyaline, thin- to slightly thick-walled, 3–5.5 µm in diam. Probasidia abundant, saccate, 14.9–32.5 × 7.5–11.4 µm. Basidia tubular-clavate, thin-walled, 4-celled, more or less straight to somewhat sinuous, 41.3–70.2 × 5.5–9.2 μm, sterigmata straight to curved, up to 5 µm long. Basidiospores germinating by germ tubes, ellipsoid to oblong ellipsoid, hyaline, thin-walled, (7.6–)8.9–13.6(–14.1) × (4.3–)4.4–8.9(–9.7) μm, L = 11.1 μm, W = 7.14 μm, Q = 1.50–1.68 (n = 120/4).

**Figure 3.**
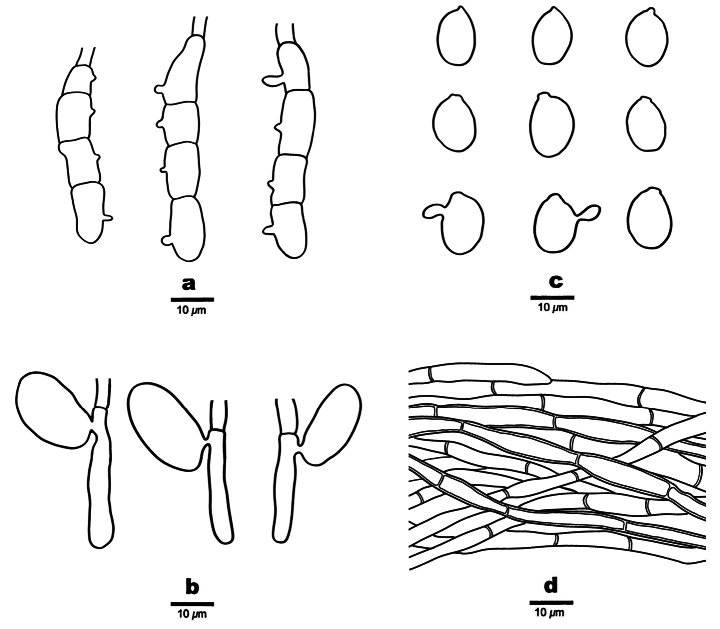
Microscopic structures of *Helicogloea candida* (holotype Wu 4197). **a**. Basidia; **b**. Probasidia; **c**. Basidiospores; **d**. Hyphae.

##### Additional specimens examined (paratypes).

China • Guangxi Auto. Reg., Baise, Leye County, Huangjingdongtiankeng National Forest Park, 24°48'42"N, 106°22'14"E, 1048 m a.s.l.; 8 September 2024, on fallen angiosperm branch, F. Wu leg., Wu 2967 (BJFC047276), Wu 2973 (BJFC047282) • Yunnan Province, Wenshan County, Jiguan Mountain, 23°19'6"N, 104°26'48"E, 1742 m a.s.l.; 11 August 2024, on fallen angiosperm branch, F. Wu leg., Wu 2924 (BJFC047233).

#### Helicogloea conidiata

Taxon classificationFungiAtractiellalesPhleogenaceae

Z.W. Zheng, Q.X. Guan & F. Wu sp. nov.

749C53C1-6639-5C3A-9C5B-6E6738B248C9

MB862385

Figs 4, 5

##### Holotype.

China • Yunnan Province, Yuxi, Xinping County, Gasa Town, Jinshan Forest Park, 23°56'32"N, 101°30'2"E, 2444 m a.s.l., 5 August 2024, on fallen angiosperm branch, F. Wu leg., Wu 2813 (BJFC047123, holotype).

**Figure 4.**
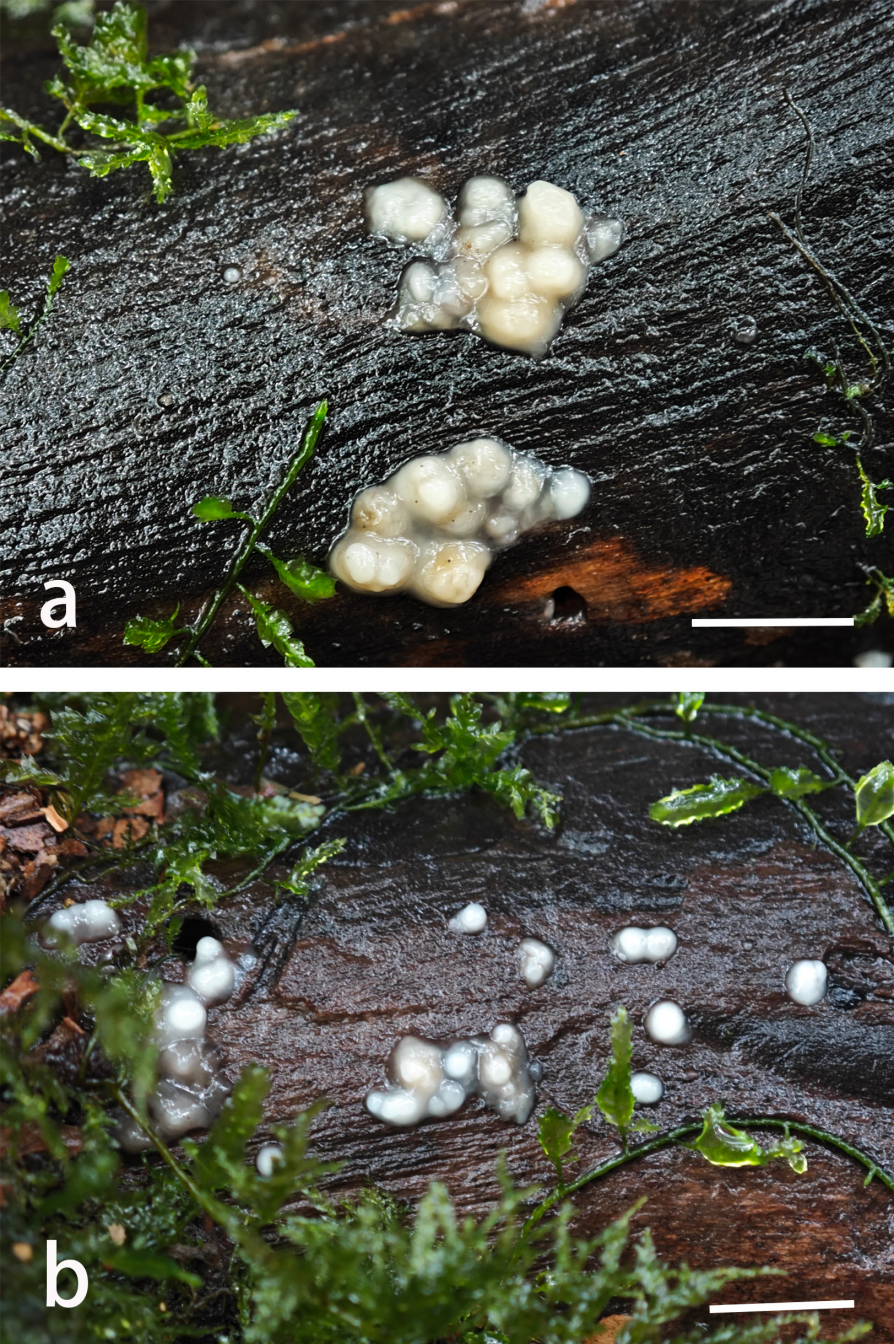
Sporodochia of *Helicogloea conidiata*. **a**. Wu 2813 (holotype); **b**. Wu 2814. Scale bars: 1 cm (**a, b**).

##### Etymology.

*Conidiata* (Lat.): refers to the new species having a sporodochial asexual morph.

##### Description.

Sporodochia soft-gelatinous, white to cream, pustulate, up to 3 mm diam. and 1 mm high when separate, sometimes coalescing when mature, turning yellowish and waxy when dry. Hyphae thin-walled, moderately branched, 2–5 µm in diam. Conidiophores irregularly branched, umbelliform. Conidiogenous cells cylindrical to slightly fusiform, straight or curved, tapering to the apex, 10–37 × 1.8–2.8 µm. Conidia subglobose to ellipsoid, hyaline, thin-walled, smooth, (3–)3.6–4.5(–4.8) × (2.8–)2.9–3.8(–4) µm, L = 4.07 μm, W = 3.23 μm, Q = 1.25–1.27 (n = 60/2).

**Figure 5.**
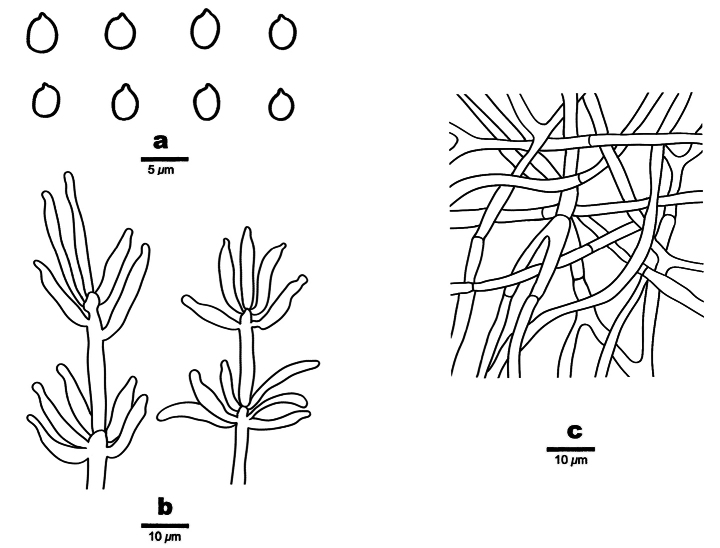
Microscopic structures of *Helicogloea conidiata* (holotype Wu 2813). **a**. Conidia; **b**. Conidiophores; **c**. Hyphae.

##### Additional specimens examined (paratype).

China • Yunnan Province, Yuxi, Xinping County, Gasa Town, Jinshan Forest Park, 23°56'32"N, 101°30'2"E, 2444 m a.s.l., 5 August 2024, on fallen angiosperm branch, F. Wu leg., Wu 2814 (BJFC047124).

#### Helicogloea sinosebacea

Taxon classificationFungiAtractiellalesPhleogenaceae

Z.W. Zheng, Q.X. Guan & F. Wu sp. nov.

986C03E6-32CD-53FF-9E77-7505EEDDE976

MB862386

Figs 6, 7

##### Holotype.

China • Hubei Province, Shennongjia National Park, Hongping Town, 31°37'26"N, 110°26'17"E, 1325 m a.s.l., 21 September 2025, on fallen angiosperm branch, F. Wu leg., Wu 4611 (BJFC064727, holotype).

**Figure 6.**
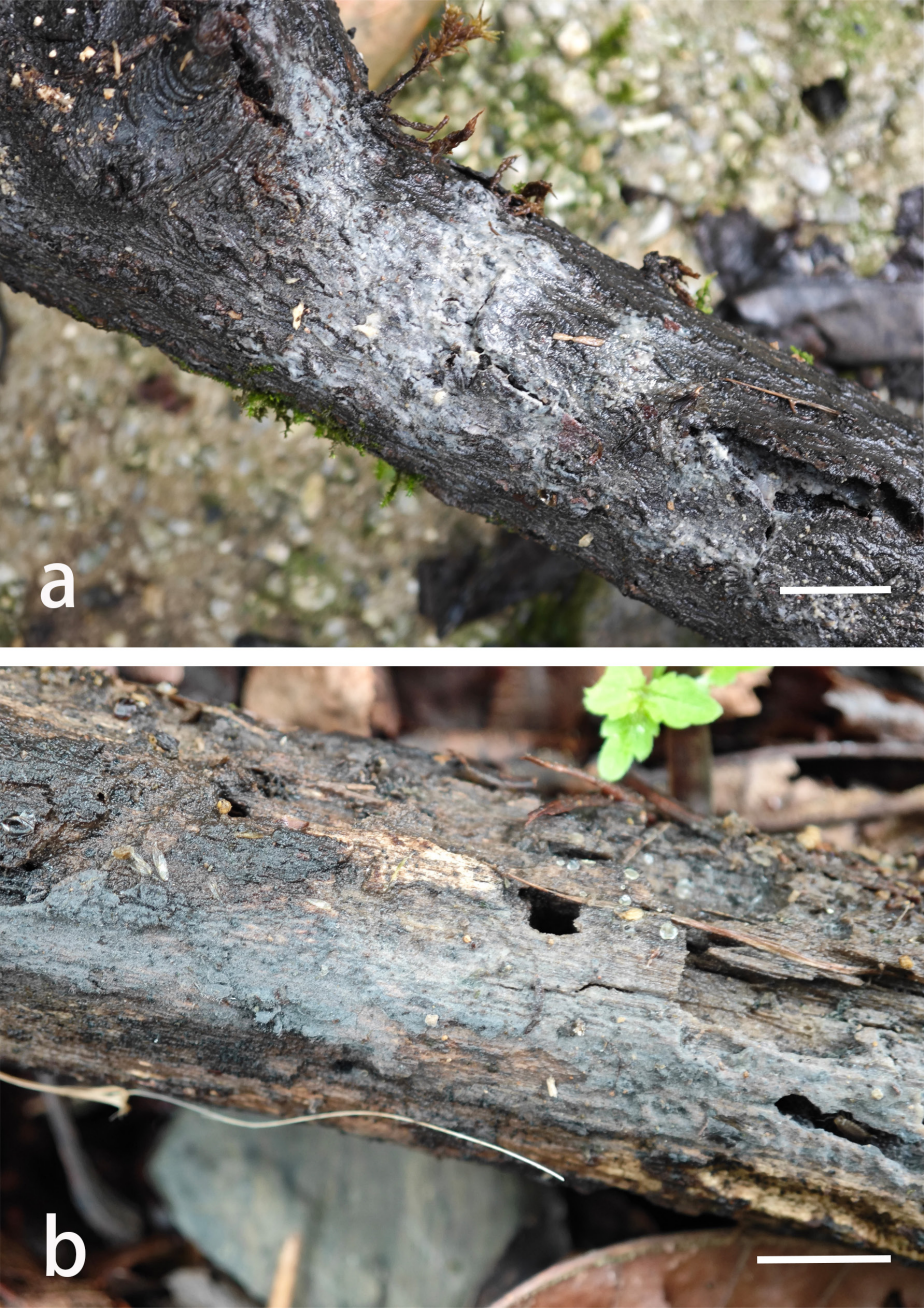
Basidiomes of *Helicogloea sinosebacea*. **a**. Wu 4611 (holotype); **b**. Wu 3224. Scale bars: 1 cm (**a, b**).

##### Etymology.

*Sinosebacea* (Lat.): refers to the new species resembling *H. sebacea* in morphology.

##### Description.

Basidiomes annual, resupinate, gelatinous when fresh, whitish or greyish, first pustulate, then fusing together and turning semi-translucent and waxy, hymenial surface often tuberculate, up to 6 cm long, 3 cm wide and 0.1 cm thick, almost invisible when dry. Hyphal system monomitic, simple septate, hyaline, thin- to slightly thick-walled, 3–6 µm in diam. Probasidia saccate, 14.1–23.3 × 5.8–10 µm. Basidia tubular-clavate, thin-walled, 4-celled, straight to slightly curved, 34.1–61.8 × 4–6.9 μm, sterigmata straight to curved, up to 7 µm long. Basidiospores germinating by germ tubes, subglobose to broadly ellipsoid, hyaline, thin-walled, 7–10(–10.8) × (4.5–)4.8–7.3(–8.7) μm, L = 8.58 μm, W = 6.35 μm, Q = 1.34–1.36 (n = 90/3).

**Figure 7.**
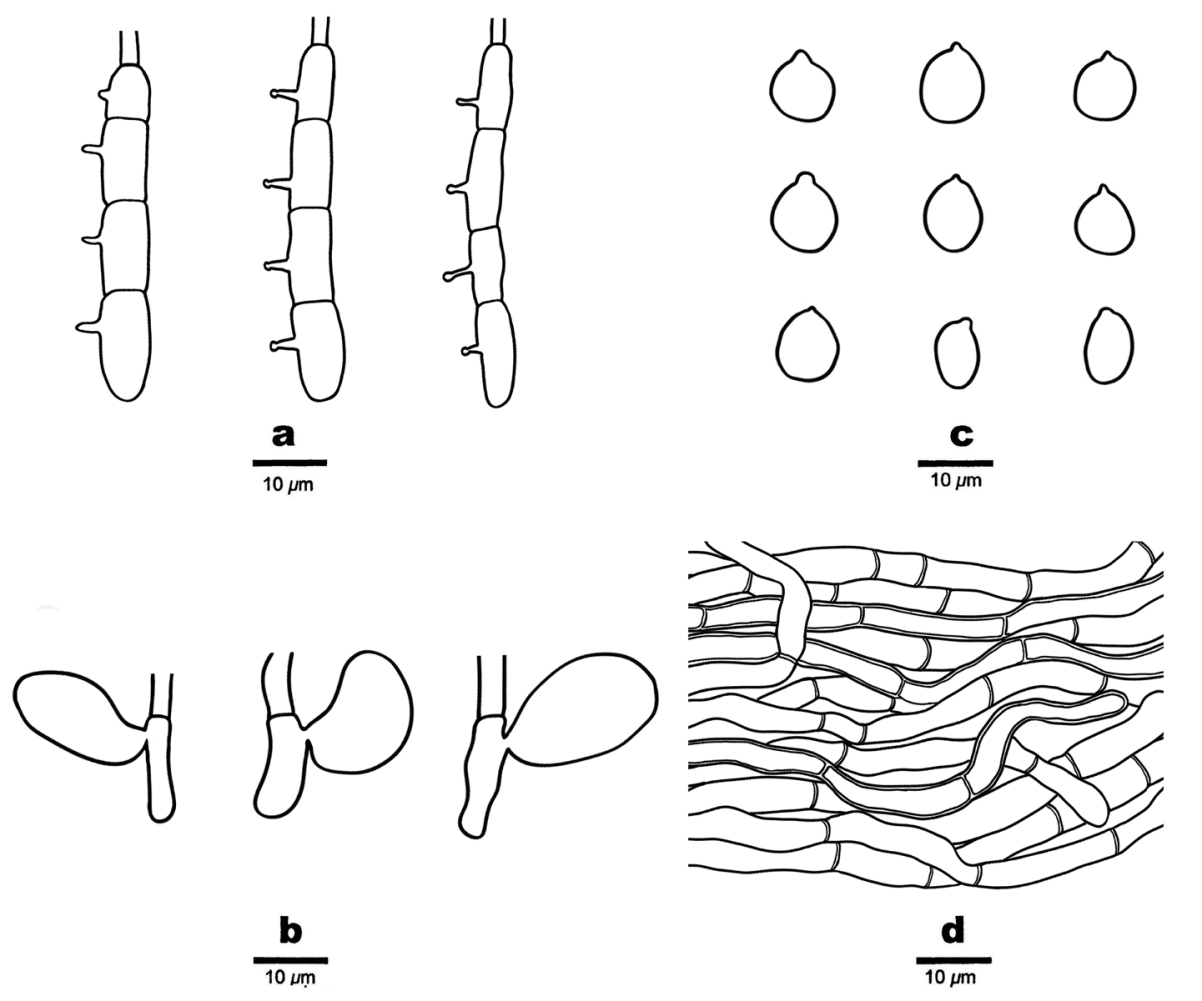
Microscopic structures of *Helicogloea sinosebacea* (holotype Wu 4611). **a**. Basidia; **b**. Probasidia; **c**. Basidiospores; **d**. Hyphae.

##### Additional specimens examined (paratypes).

China • Zhejiang Province, Hangzhou, Linan County, West Tianmu Mountain Scenic Spot, 30°18'53"N, 119°26'20"E, 327 m a.s.l., 23 June 2025, on fallen angiosperm branch, F. Wu leg., Wu 3224 (BJFC063340); 30°19'54"N, 119°26'14"E, 564 m a.s.l., 14 September 2025, on fallen angiosperm branch, F. Wu leg., Wu 4211 (BJFC064327).

## Discussion

In this study, three new species of *Helicogloea* from south China are described, contributing to a deeper understanding of *Helicogloea* diversity in China. Morphologically, *Helicogloea sinosebacea* is similar to *H. septifera* Spirin & Malysheva by sharing gelatinous and whitish or greyish basidiomes when fresh, but *H. septifera* differs by its longer basidia (56–101 × 6.8–9.0 μm vs. 34.1–61.8 × 4–6.9 μm) and longer basidiospores (11.1–16.2 × 6.2–9.1 μm vs.7–10 × 4.8–7.3 μm; Spirin et al. (2018)). Phylogenetically, *H. sinosebacea* formed an independent and well-supported lineage closely related to *H. sebacea* (Fig. 1). The two species share whitish or greyish basidiomes, but *H. sebacea* has only waxy basidiomes, broadly cylindrical to narrowly ellipsoid basidiospores and smaller basidia (7–48 × 5.2–6.8 μm vs. 34.1–61.8 × 4–6.9 μm; Spirin et al. (2018)). In addition, the ITS sequences show approximately 11% differences between the two taxa.

*Helicogloea candida* is morphologically similar to *H. insularis* Spirin & K.H. Larss. and *H. hangzhouensis* by having gelatinous and postulate basidiomes, but *H. insularis* differs by its thinner basidiomes (0.05–0.1 mm thick vs. 0.1 cm), cylindrical basidiospores, smaller probasidia (13–24 × 4.5–6.0 µm vs. 14.9–32.5 × 7.5–11.4 µm), and distribution in Europe (Malysheva et al. 2020). *Helicogloea hangzhouensis* differs by its longer basidiospores (12.1–15.1 × 6.2–8.6 μm vs. 8.9–13.6 × 4.4–8.9 μm) and longer probasidia (30.2–39.5 × 8.1–10.5 µm vs. 14.9–32.5 × 7.5–11.4 µm; Liu et al. (2025)). Phylogenetically, *H. candida* formed one distinct lineage most related to *H. aseptata*. However, *H. aseptata* differs from *H. candida* by semi-translucent vernicose crust basidiomes when dry and longer basidiospores (10.3–13.8 × 5.8–8.1 μm vs. 8.9–13.6 ×4.4–8.9 μm; Malysheva et al. (2020)). In addition, the ITS sequences similarity between *H. aseptata* (Spirin 12172) and *H. candida* is only 86.7%.

*Helicogloea conidiata* was discovered in the subtropical region of China and the two specimens presented only sporodochial asexual morphs. Kirschner (2004) noted that sporodochial asexual morphs had been detected in *Helicogloea*. The species *H. eburnea*, described by Spirin et al. (2018), also presents a sporodochial asexual morph. In our phylogeny, *H. conidiata* is closely related to *H. eburnea* and the ITS sequences differ by 4% between the two taxa. Morphologically, *H. eburnea* differs from *H. conidiata* by its white semi-translucent sporodochia, narrower conidia (2.9–3.7 × 2–2.6 µm vs. 3.6–4.5 × 2.9–3.8 µm) and distribution in Africa (Spirin et al. 2018).

Although the taxonomy of the genus *Helicogloea* has been studied in recent years, several species within the genus still lack molecular data. Spirin et al. (2018) reported that 10 species of *Helicogloea* s. str. have been identified in Europe and all appear to be host-specific: seven occur exclusively on angiosperm wood and only two on conifers. The three new species described here were all found on angiosperm wood and showed similarities to other wood-decaying fungi documented by Spirin et al. (2018).

## Supplementary Material

XML Treatment for Helicogloea candida

XML Treatment for Helicogloea conidiata

XML Treatment for Helicogloea sinosebacea

## Additional information

### Artificial Intelligence (AI) use

The authors accept full responsibility for the content of the manuscript, including the disclosure of any use of AI.

No AI tools were used in the preparation of this manuscript.

### Author ORCIDs

Z.-W. Zheng https://orcid.org/0009-0006-8442-408X

Q.-X. Guan https://orcid.org/0000-0002-7072-080X

S.-B. Liu https://orcid.org/0000-0002-2275-3922

F. Wu https://orcid.org/0000-0002-1455-6486

### Data availability

All of the data that support the findings of this study are available in the main text or Supplementary Information.

## Supplementary materials

Supplementary material 1 32513treebase Data type nxs https://binary.pensoft.net/file/1630804This dataset is made available under the Open Database License (http://opendatacommons.org/licenses/odbl/1.0/). The Open Database License (ODbL) is a license agreement intended to allow users to freely share, modify, and use this Dataset while maintaining this same freedom for others, provided that the original source and author(s) are credited. Zi-Wei Zheng, Qian-Xin Guan, Shu-Bin Liu, Fang Wu
